# Phosphoprotein Associated with Glycosphingolipid-Enriched Microdomains Differentially Modulates Src Kinase Activity in Brain Maturation

**DOI:** 10.1371/journal.pone.0023978

**Published:** 2011-09-06

**Authors:** Sabine Lindquist, Diana Karitkina, Kristina Langnaese, Anita Posevitz-Fejfar, Burkhart Schraven, Ramnik Xavier, Brian Seed, Jonathan A. Lindquist

**Affiliations:** 1 Department of Neurology, Hannover Medical School, Hannover, Germany; 2 Leibniz Institute for Neurobiology, Magdeburg, Germany; 3 Institute for Biochemistry and Cell Biology, Otto-von-Guericke University, Magdeburg, Germany; 4 Institute of Molecular and Clinical Immunology, Otto-von-Guericke University, Magdeburg, Germany; 5 Department of Immune Control, Helmholtz Centre for Infection Research, Braunschweig, Germany; 6 Center for Computational and Integrative Biology, Massachusetts General Hospital, Harvard Medical School, Boston, Massachusetts, United States of America; University of South Florida College of Medicine, United States of America

## Abstract

Src family kinases (SFK) control multiple processes during brain development and function. We show here that the phosphoprotein associated with glycosphigolipid-enriched microdomains (PAG)/Csk binding protein (Cbp) modulates SFK activity in the brain. The timing and localization of PAG expression overlap with Fyn and Src, both of which we find associated to PAG. We demonstrate in newborn (P1) mice that PAG negatively regulates Src family kinases (SFK). P1 *Pag1* ^-/-^ mouse brains show decreased recruitment of Csk into lipid rafts, reduced phosphorylation of the inhibitory tyrosines within SFKs, and an increase in SFK activity of >/ = 50%. While in brain of P1 mice, PAG and Csk are highly and ubiquitously expressed, little Csk is found in adult brain suggesting altered modes of SFK regulation. In adult brain *Pag1*-deficiency has no effect upon Csk-distribution or inhibitory tyrosine phosphorylation, but kinase activity is now reduced (−20–30%), pointing to the development of a compensatory mechanism that may involve PSD93. The distribution of the Csk-homologous kinase CHK is not altered. Importantly, since the activities of Fyn and Src are decreased in adult *Pag1* ^-/-^ mice, thus presenting the reversed phenotype of P1, this provides the first *in vivo* evidence for a Csk-independent positive regulatory function for PAG in the brain.

## Introduction

Src family kinases (SFK) play key roles in regulating the cell cycle, differentiation, cell-cell contact, and receptor activation in various cell types [1]. The expression pattern and developmental regulation of SFKs in the central nervous system are partly overlapping and analysis of knockout mice demonstrated considerable redundancy among the SFKs, but also some unique functions. Specifically, *Fyn*-deficient mice show alterations in the neuronal architecture of the hippocampus and are hypomyelinated [2]–[4]. Functional deficits include impaired long-term potentiation and spatial learning, increased anxiety, and a higher sensitivity to ethanol [2], [5], [6]. Interestingly, *Fyn*-knockout mice compensate by upregulating the activity of Src, specifically in the Triton-X-insoluble fractions [7], [8]. The importance of Fyn and Src redundancy is highlighted in *Src* ^-/-^/*Fyn* ^-/-^ double-knockout mice of which 85–90% die perinatally and *Src* ^-/-^-*Fyn* ^-/-^-*Yes* ^-/-^-triple-mutants which all die early in gestation [7], [9].

Src family kinases are targeted to the membrane by lipid modifications of their N-terminus and their kinase activity is regulated by phosphorylation. While phosphorylation of tyrosine 418 (Y418 human Src) within the kinase domain is associated with activation, phosphorylation of tyrosine 529 (Y529 human Src) in the regulatory C-terminus allows intramolecular binding to the SH2-domain, resulting in a closed-inactive conformation [10]. Csk was the first kinase identified to phosphorylate Y529 [11]. In *Csk*-knockout mice the activity of Src, Fyn, and Lyn is constitutively enhanced [12]. The generalized dysregulation of Src kinase activity causes an embryonically lethal phenotype with gross neural tube defects and dramatic neuronal cell death [12], [13]. Csk is highly expressed during development, but expression in brain rapidly declines after birth. Its homologue, CHK (Csk-homologous kinase) also phosphorylates Y529 and shows the opposite expression pattern, increasing in abundance in adult brain. Both Csk and CHK lack the lipid modification necessary for association to the membrane compartment where Src kinases reside. Brdicka et al. [14] and Kawabuchi et al. [15] both identified PAG (phosphoprotein associated with glycosphingolipid-enriched microdomains)/ Cbp (Csk-binding protein) as a molecule that recruits Csk to the plasma membrane and into lipid rafts. The function of PAG was first characterized in human T-lymphocytes where Fyn was shown to constitutively bind PAG [14]. Phosphorylation of Y314 in murine PAG (Y317 in human) by SFK allows the SH2 domain of Csk to bind and thus recruits Csk into the lipid rafts where it inhibits Src kinases. Upon T-cell receptor activation, PAG becomes rapidly dephosphorylated, Csk binding is lost, and Fyn is activated via dephosphorylation of Y529 and autophosphorylation of Y418. Active Fyn then rephosphorylates PAG, recruiting Csk, and thus terminating the signal (reviewed in [16]). This model could subsequently be extended to the control of Lyn kinase activity in Fc_ε_RI signaling [17], [18] and Src and Yes activity upon EGF-receptor stimulation [19] as well as to the regulation of adhesion and migration of embryonal fibroblasts [20].

The PAG-Csk-complex was originally isolated from the brain of newborn rats where Fyn is primarily responsible for PAG phosphorylation [15], [20], and a role in brain development is suggested by our recent study in a mouse model of muscular dystrophy. These *α2-Laminin*-deficient mice show delayed oligodendrocyte maturation associated with an increase in phosphorylation of the inhibitory Y529 in Fyn, which correlates well with an increased expression of PAG and Csk in cortices of 3 weeks old mice [21].

In the current study, we demonstrate that PAG is necessary for the recruitment of Csk and thus inhibition of SFK activity in postnatal brain, but functions Csk-independently as a positive regulator of SFK in brains of adult mice.

## Materials and Methods

### Antibodies and reagents

Rabbit phosphor-specific anti-Src (pY418), recognizing both Y423 in mouse Src and Y419 in mouse Fyn, as well as anti-Src (pY529), recognizing both Y534 in mouse Src and Y530 in mouse Fyn due to the highly conserved sequence of these residues [22] were purchased from BioSource, rabbit anti-Csk (C-20) and anti-Ctk from Santa Cruz, mouse-anti-Csk (clone 52) and mouse anti-CHK from BD BioSciences, mouse anti-Src (clone GD11) from Upstate (Lake Placid, NY), rabbit anti-PSD93 from Alomone Labs Ltd., rabbit anti-c-Src (Y215) from ECM Biosciences, and mouse anti-β-actin (clone AC-15) from Sigma. Mouse-anti-phosphotyrosine (4G10) antibodies were produced in our institute from the hybridoma cells. Secondary antibodies goat-anti-mouse-HRP and goat-anti-rabbit-HRP were obtained from Dianova.

Rabbit polyclonal anti-PAG (residues 97-432) and mouse anti-Fyn (Fyn 02) were kindly provided by Vaclav Horejsi (Prague, CZ). Anti-phospho-PAG (pY317/pY314) was developed by Burkhart Schraven [23]. Rabbit polyclonal anti-PAG (Ig452) against the peptide CGDLQQGRDVTRL was developed in collaboration with ImmunoGlobe (Berlin, Germany). The antibodies against PAG, Fyn and Src used here recognize the respective proteins phospho-independently (Fig. S1). Chemicals were purchased from Sigma unless stated otherwise.

### *Pag1*-knockout mice

Mice with a deletion in the *Pag1* gene were generated using modified bacterial artificial chromosome (BAC) technology as previously described [24]. In brief, we obtained BAC clones spanning the *Pag1* locus from Research Genetics (Invitrogen Life Technologies). Two short sequences flanking exons 6 were cloned into the 5′ and 3′ insertion sites of the selection cassette of the pSKY replacement vector. BAC host cells were transformed with the pBADred plasmid, which helped produce electroporation-competent cells. The linear fragment released from the pSKY backbone was then electroporated into the BAC host, and the transformants were selected for simultaneous resistance to chloramphenicol (from the BAC backbone) and zeocin (from the insert). The resulting mutant BAC is shown in Fig. S2A.

As shown in Fig. S2B, PAG1/PAG2/Pzeo primers (pag1: ttctttcagaagacagcacgctg; pag2: gcgtccaccggtcccttctgcag), identify a predicted 473-bp PCR product in the mutant BAC confirming 5′ targeting. As predicted, the Psv/PAG1/PAG2 primers identify a 745-bp PCR product in the mutant BAC and 581-bp PCR product in the wild-type BAC. Restriction digestion with BamHI and HindIII confirms the identity of WT and mutant BACs (Fig. S2C). Fluorescence in situ hybridization was performed as described previously and confirmed targeting in clones 7 and 35 (Fig. S2D). *Pag1* ^–/–^ mice from clone 7 were born in the expected Mendelian frequency of 25% in *Pag1* ^+/−^ crossings with a male/female ratio of 1∶1. In *Pag1* ^-/-^ breedings the male/female ratio was 1∶0.94 (66 animals analyzed). All experiments were performed on tissue from euthanized animals in accordance with the German National Guidelines for the Use of Experimental Animals (Animal Protection Act, Tierschutzgesetz, TierSchG, in particular paragraph 7 and 8). Animals were handled in accordance with the European Communities Council Directive 86/609/EEC. All possible efforts were made to minimize animal suffering and the number of animals used.

### *In situ*-hybridization

*In situ*-hybridization was carried out on 18- µm-thick cryosections of mouse brains as previously described [25], [26]. For the antisense-probe, a pBluescript II SK plasmid containing the complete mouse PAG cDNA (accession number AF250192) [14] was linearized with BamHI (internal BamHI site), *in vitro* transcribed with T7 RNA polymerase yielding a cRNA of ∼900 bases. To produce sense cRNA for control the plasmid was linearized with Hind III, yielding a ∼800 bases long antisense cRNA (internal HindIII site); the cRNA was *in vitro* transcribed with T3 RNA polymerase. The specific activity of the probes was ∼10^8^ cpm/µg. Radioactivity was adjusted to 3,3×10^6^ cpm/100 µl hybridization buffer and used immediately. The slides were dipped, developed, and counterstained Mayer's hematoxylin solution and eosin as previously described.

### RNA extraction

RNA was isolated from whole brains of C57Blc6 mice at the indicated age (except E12: whole embryo head) using peqGOLD TriFast according to the manufacturer's instructions (Peqlab, Erlangen, Germany). RNA samples were dissolved in water and quantified spectrophotometrically at 260 *nm*. After treatment with TURBO-DNase (TURBO DNA-freeTM, Ambion Inc.) according to the manufacturer's instructions, 2 µg of total RNA were transcribed into cDNA using RevertAidTM H Minus M-MuLV Reverse Transcriptase and the RevertAidTM H Minus First Strand cDNA Synthesis Kit according to manufacturer's instructions (Fermentas).

### RT-PCR

Primer sequences were designed to span intron regions to insure that no false positive PCR fragments would be generated from pseudogenes in the contaminating genomic DNA. The following primers were used:

PAG-r (AGTTCCTTGTCATACAAGTCTCG),

PAG-f (CCACTTCTGCCTTGAAGGAGCTTC),

Fyn-r (TGGCACAGGAGCAGCTATTTA C),

Fyn-f (GAGCTGGTCACCAAAGGAAGAG),

Lyn-r (TCTGACTGTGGTCCCATTGAGC),

Lyn-f (GATTGTCACCTATGGGAAGATTC),

Csk-r (ACAGGCAGGTACAGAGGCAAG),

Csk-f (CGTTCGGTGCTAGGTGGAGAC),

Ctk-r (ACTATTTTGCGGAAGGGTGGTC),

Ctk-f (CGTGCTCTTGTGAGCACCTCTC),

GAPDH-r (CTTACTCCTTGGAGGCCATG),

GAPDH-f (TTAGCACCCCTGGCCAAGG).

For each gene, the linear amplification range was optimized. Reactions were performed on a PCR cycler (GeneAmp, PCR System 9700, PE Applied Biosystems) using cDNA (1.1 ng), oligonucleotide primer (10 pmol each), of each dNTP (200 µM each), SAWADY Taq-DNA-Polymerase (2 units, PEQLAB) and 1X reaction buffer S in a 50 µl volume. Amplification started with a 95°C denaturation for 5 min, followed by the optimized number of cycles of 95°C/30 sec, 55°C/30 sec, 72°C/1 min. The PCR products were visualized by electrophoresis on 2% agarose gels in TBE buffer (89 mM Tris-base pH 7.6, 89 mM boric acid, 2 mM EDTA) with 10 µg/ml ethidium bromide and photographed using a UV light box.

### GEM-Preparation

To isolate glycosphingolipid-enriched microdomains, C57Blc6 mice brains were homogenized with ULTRA-TURRAX T25 (Janke & Kunkel GmbH, IKA-Labortechnik) and lysed at a concentration of 1 g/3 ml in ice-cold Brij 58-containing lysis buffer (3% Brij 58, 50 mM Hepes pH 7.4, 100 mM NaCl, 1 mM AEBSF (AppliChem GmbH), 5 mM EDTA, 1 mM sodium orthovanadate, 50 mM sodium fluoride, 10 mM sodium pyrophosphate) for 10 min on ice. Lysates were then subjected to sucrose gradient centrifugation as previously described [14]. GEM-containing fractions were detected by dot blot probed with biotinylated cholera toxin subunit B (Sigma) and streptavidin-HRP (Dako A/S, Glostrup, Denmark).

### Immunoprecipitation and Western blot analysis

Brain homogenates in GEM buffer were incubated with 1% N-dodecyl β-D-maltoside [lauryl maltoside] (Calbiochem) for 30 min on ice. After incubation, lysates were centrifuged for 15 min at 15,700xg. For direct Western blot analysis supernatants were heated in 1x reducing sample buffer for 5 min at 95°C. For immunoprecipitation, the postnuclear lysate was immunoprecipitated with the respective antibodies/-sera as previously described [14]. Gel electrophoresis of immunoprecipitates and lysates as well as ECL-Western blotting have also been described [14].

### *In vitro*-kinase assays

Fyn and Src were immunoprecipitated from pooled GEM-containing fractions following incubation with 2% lauryl-maltoside for 30 min on ice and centrifugation for 15 min at 15,700xg at 4°C, using either 0.5 µl Fyn 02 and 30 µl protein A sepharose; or 4 µl anti-Src, 1 µl rabbit-anti-mouse (Dako) and 30 µl protein A sepharose in the presence of 1 mg/ml BSA for 18 h at 4°C with gentle rotation. Immunoprecipitates were washed and subjected to the kinase reaction as previously described [14]. Samples were separated by 10% SDS-PAGE, dried and exposed to film at –70°C with intensifying screen and on Phosphoimager plates. Quantification from films and phosphoimager plates yielded the same results.

### PAG constructs

To express N-terminally Flag-tagged PAG, an oligonucleotide containing the CD8a leader sequence (aa 1–21) and FLAG tag was ligated into pGEM-5Zf vector using the Aat II-Nco I sites. The PAG sequence was then inserted at the Nco I-Not I site. The Flag-tagged PAG was excised from the pGEM vector and inserted into pEF-IRES using Sma I-Not I ligation. The Nhe I site within the CD8-Flag tag was used for screening the colonies. Sequence integrity was verified by sequencing (GATC Biotech). Stop mutants were generated using the Quick-change site directed mutagenesis kit II (Stratagene) following the manufacturers instructions. The primer sequences used were:

STOP1-f (ACAGCATAGTGGGGACCATTAGAACCTGATGAACGTGC),

STOP1-r (GCACGTTCATCAGGTTCTAATGGTCCCCACTATGCTGT),

STOP2-f (GATCTGCTGGATTCCTAGGACAGCACAGGGAAAC),

STOP2-r (GTTTCCCTGTGCTGTCCTAGGAATCCAGCAGATC),

STOP3-f (CGTCAAAGTGTTAATGTATAGAGTATCCTTGG AAATTC),

STOP3- r (GAATTTCCAAGGATACTCTATACATTAACACTTTGACG),

STOP4-f (GCTACTGTTAAAGACTTCTAAAAAACTCCAAACAGCAC),

STOP4-r (GTGCTGTTTGGAGTTTTTTAGAAGTCTTTAACAGTAGC).

## Results

### Developmental expression of PAG in brain

**Figure 1:**
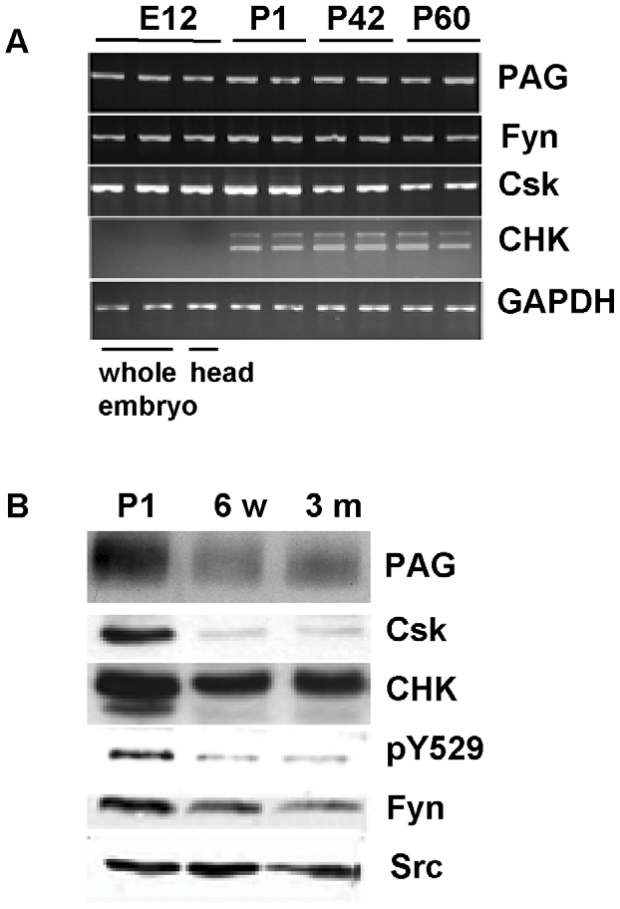
Expression of PAG during brain development. **A**) Total RNA was isolated from whole C57Bl6 mouse brains (head or whole embryo for E12) of different ages as indicated. The expression of PAG-mRNA and functionally related genes was analyzed following an RT-reaction and PCR using gene-specific primers. CHK-RT-PCR produced a band at the expected molecular weight (460 bp) as well as a much weaker band at 560 bp. Sequencing revealed that the latter band represented an incompletely spliced product including intron 10. One representative out of three similar experiments is shown. **B**) Expression of PAG and functionally related proteins was analyzed by Western blot from whole brain lysate of postnatal C57Bl6 mice of different ages as indicated. PAG was detected here with the antiserum against the whole cytoplasmic domain. Detection with the anti-PAG peptide antiserum Ig452 resulted in a similar signal. As all structural proteins tested showed differential expression during development, equal loading was assured by determination of protein concentration (50 µg/lane) and controlled for by Coomassie staining of the gels (not shown). A double band for CHK at P1 was consistently shown with both a polyclonal anti-CHK-antiserum and a monoclonal antibody against this protein and may represent two different isoforms or phosphorylated forms. A similar double band has previously been shown in Western blot [55]. One representative out of four similar experiments is shown.

The function of PAG was first defined as a Csk-binding protein. However, Csk expression in brain is strongly developmentally regulated and expression in adult brain was reported to be limited to the olfactory bulb [27]. Therefore, we tested the mRNA expression of PAG as well as candidate interaction partners at different stages in mouse brain development. Time points were chosen to reflect early embryonal Csk expression at a time when the embryos head can first be separately analyzed (E12), the peak of Csk expression (P1), myelinating juvenile brain (P42), and adult brain (P60). RT-PCR analysis (Fig. 1A) shows that PAG is expressed at all of these time points. We confirm that Csk expression peaks around P1, but contrary to previous reports [27] we show considerable expression in juvenile and adult brain. In agreement with previous work, we demonstrate that the expression of the Csk-homologous kinase CHK is regulated opposite to that of Csk with expression detected from P1 onwards, while Fyn is expressed throughout. We now focused our analysis on postnatal and adult brains as these time points showed the strongest differences in Csk-expression, as well as in 6 week old brains, where Fyn activity is important for proper myelination [4]. Western blot analysis (Fig. 1B) shows that PAG protein expression is strongest at P1 whereas it is decreased in 6 week and 3 month old brains. Csk protein expression is strong at P1, but hardly detectable at 6 weeks and 3 months, while CHK expression remains strong throughout. Fyn expression is well visible at P1 and decreases slightly at the later time points, while expression of Src remains fairly constant. Interestingly, phosphorylation of Y529, the negative regulatory site within SFKs phosphorylated by Csk and CHK, is very high at P1 and is much weaker at the later time points. This difference cannot simply be explained by changes in the expression levels of Fyn and Src, but fits rather well with the higher expression of Csk and CHK at P1.

### Localization of PAG expression in postnatal and adult mouse brain

**Figure 2:**
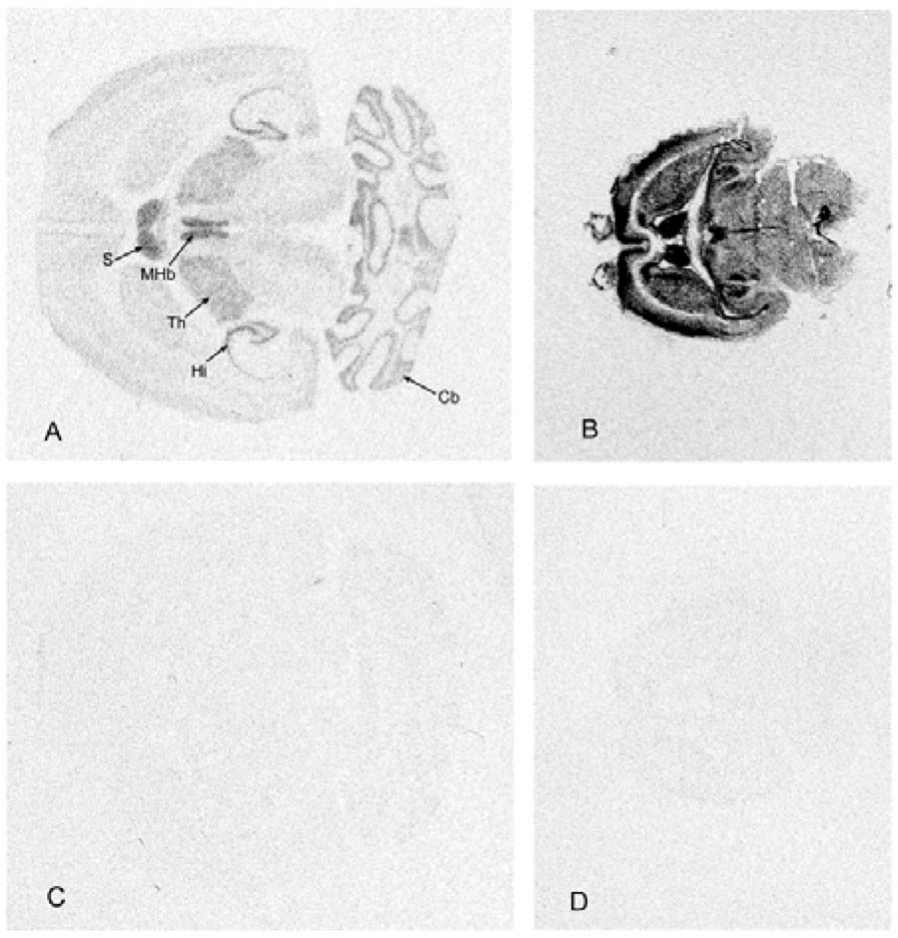
*In situ* distribution of PAG transcripts. Film autoradiographs of horizontal mouse brain sections showing PAG mRNA distribution in **A**) adult and **B**) postnatal (P1) mice. The respective sense controls are shown below (**C, D**). In the adult brain most prominent mRNA expression was detectable in septum (S), medial habenula (MHb), thalamic nuclei (Th), hippocampus (Hi), and cerebellum (Cb). One representative out of two similar experiments is shown.

**Figure 3:**
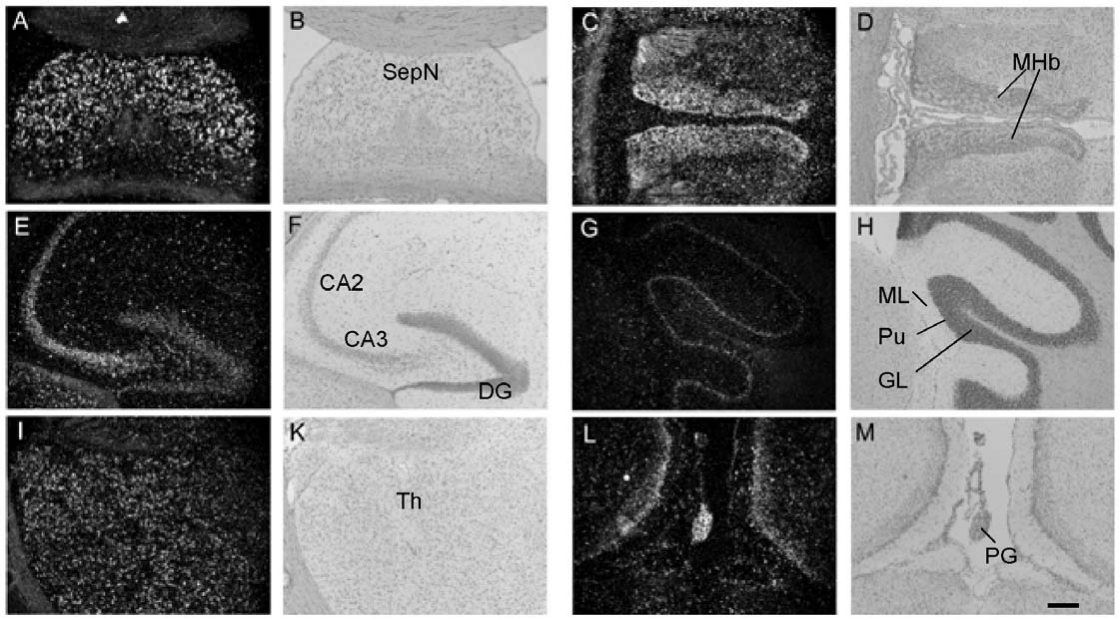
Localization of PAG transcripts in specific regions of adult brain. Representative dark-field micrographs (**A, C, E, G, I, L**) illustrating the hybridization and the respective bright-field (**B, D, F, H, K, M**) micrographs in adult mouse brain. Pictures are taken from horizontal sections, with the exception of **L** and **M**, which are photographed from frontal sections. **A, B**) Septal nuclei (SepN), **C, D**) habenula (MHb), **E, F**) hippocampus: CA2 and 3 and dentate gyrus (DG), **G, H**) cerebellum (ML: molecular layer, Pu: Purkinje cell layer, GL: granular cell layer), **I, K**) thalamus (Th), **L, M**) pineal gland (PG). Scale bar 200 µm.

As postnatal and adult mouse brain showed striking differences in the levels of Csk and, to a lesser extent, PAG expression, we next studied whether the localization of PAG-mRNA expression differed at these time points. Measuring the mean density in autoradiographs we found that overall expression of PAG-mRNA in adult brains was 50% of that in P1 brains (Fig. 2), correlating well to the difference in protein expression (Fig. 1B). At both ages, a neuronal expression pattern dominated. At P1, PAG-mRNA expression was high in most areas of gray matter, namely cerebral cortex, hippocampus, septal nuclei, habenula, olfactory bulb, and striatum. In adult mouse brain PAG-mRNA expression was concentrated in the limbic system and in cerebellum. The highest expression levels were found in the hippocampus, particularly in the CA3 area and the dentate gyrus (Fig. 3), in the septal nuclei, medial habenula, and the pineal gland. Furthermore, cerebellar cortex showed a striking expression in the Purkinje cell layer. Intermediate expression levels were found in cerebral cortex, thalamus, and olfactory bulb, particularly in the mitral cell layer and inner granule cell layer. Brain stem showed a lower PAG expression and no particular nuclei could be delineated. In spinal cord, PAG-mRNA was concentrated in gray matter and expression showed a gradient towards higher expression in the dorsal horns (not shown). White matter showed a low expression of PAG-mRNA, which nevertheless exceeded background levels.

### PAG binding partners

**Figure 4:**
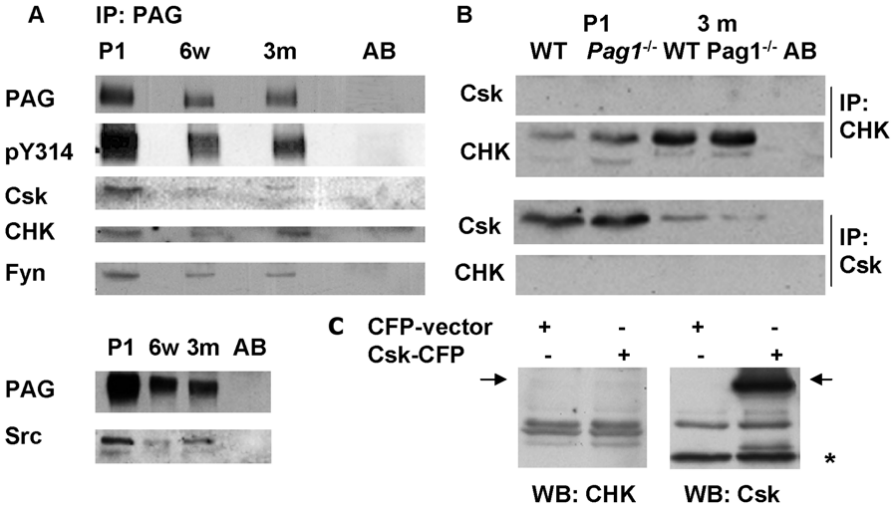
Binding partners of PAG in postnatal and adult brain. **A**) PAG immunoprecipitates from whole brain lysates of newborn, 6 month old, and adult mice were analyzed by Western blot. Each association was shown in at least three independent experiments. **B**) Csk and CHK were immunoprecipitated from lysates of adult mouse brain. Western blot analysis with CHK and Csk-antibodies, respectively, reveals no cross-reactivity. **C**) HEK-293 cells were transfected with a mouse-Csk-CFP construct versus vector containing CFP alone. Over-expression is demonstrated with the anti-Csk-antibody. There is no cross-reactivity of the anti-CHK-antibody with the over-expressed Csk. Csk-CFP is indicated with an arrow, endogenous Csk with an asterisk.

Within the immune system, the binding and recruitment of Csk into lipid rafts, thereby regulating the activity of Src family kinases, has been defined as a crucial function of PAG. We show here, that Y314 of PAG, the residue critical for Csk binding, is phosphorylated with little difference in developing and adult brain (Fig. 4A). Given the low expression of Csk and the specific localization of PAG expression in adult brain, we tested whether Csk can still be found associated at this time point. We could readily reproduce Csk-binding to PAG at P1; at 6 weeks and 3 months very little Csk can be co-precipitated. As the SH2 domain of CHK is highly homologous to Csk and CHK expression, contrary to Csk, increases during brain maturation, we tested whether CHK can also associate to PAG. Using both a polyclonal anti-CHK-serum and a monoclonal anti-CHK-antibody, we could co-precipitate CHK with PAG in developing and adult brain. As both Csk and CHK run at similar molecular weights and the overall homology between both proteins is ∼50% (this is also true of the C-terminal 100 amino acids to which the antibodies were generated) the specificity of our antibodies was tested in Csk-and CHK immunoprecipitates, where no cross-reactivity was shown (Fig. 4B). Furthermore, both the anti-CHK-antiserum and the anti-CHK–antibody did not cross-react with mouse Csk overexpressed in HEK-293 cells. Thus, we can demonstrate a specific association of CHK to PAG in mouse brain.

PAG has been shown to be phosphorylated by Src kinases, particularly Fyn. In brains of postnatal *Fyn*-deficient mice a reduced total tyrosine phosphorylation of PAG was shown [20], however, the binding of Fyn to PAG in CNS cells or tissue has not been demonstrated to date. We show that in postnatal, 6 week, and three month old brains Fyn is associated to PAG. Moreover, we demonstrate the binding of Src in developing and mature brain (Fig. 4A).

We could not detect an association of Lyn to PAG in our whole brain extracts (data not shown), which had been demonstrated in mast cells [18]. However, this may be explained by the lower expression levels of Lyn during development and its specific concentration in the cerebellum.

### PAG contributes to the recruitment of Csk to lipid rafts in postnatal brain

**Figure 5:**
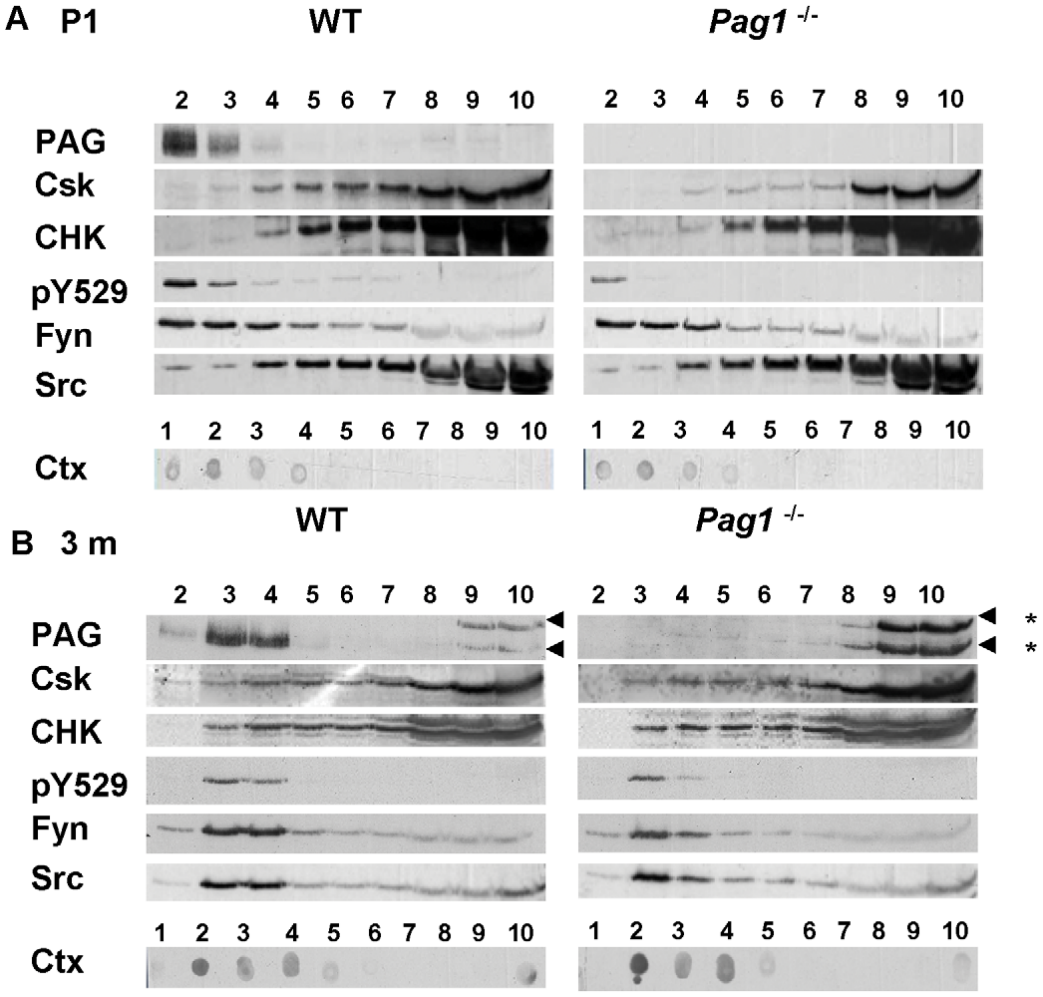
Reduced Csk-recruitment to lipid rafts in P1- *Pag1* ^-/-^ mice. Whole brains from **A**) P1 wild type C57Bl6 (WT) and *Pag1* ^-/-^ mice or **B**) 3 month old WT/ *Pag1* ^-/-^ littermate pairs were lysed in GEM-lysis buffer and subjected to sucrose-density centrifugation. Fractions were analyzed by Western blotting. PAG was detected using the Ig452 antiserum. Lipid raft fractions were identified by binding of cholera toxin subunit B (Ctx) binding to GM1-lipids in a dot blot from the fractions. * Arrowheads indicate non-specific bands in 3 months old *Pag1* ^-/-^ and wild type brain extracts, see also Fig. 6A.

**Figure 6:**
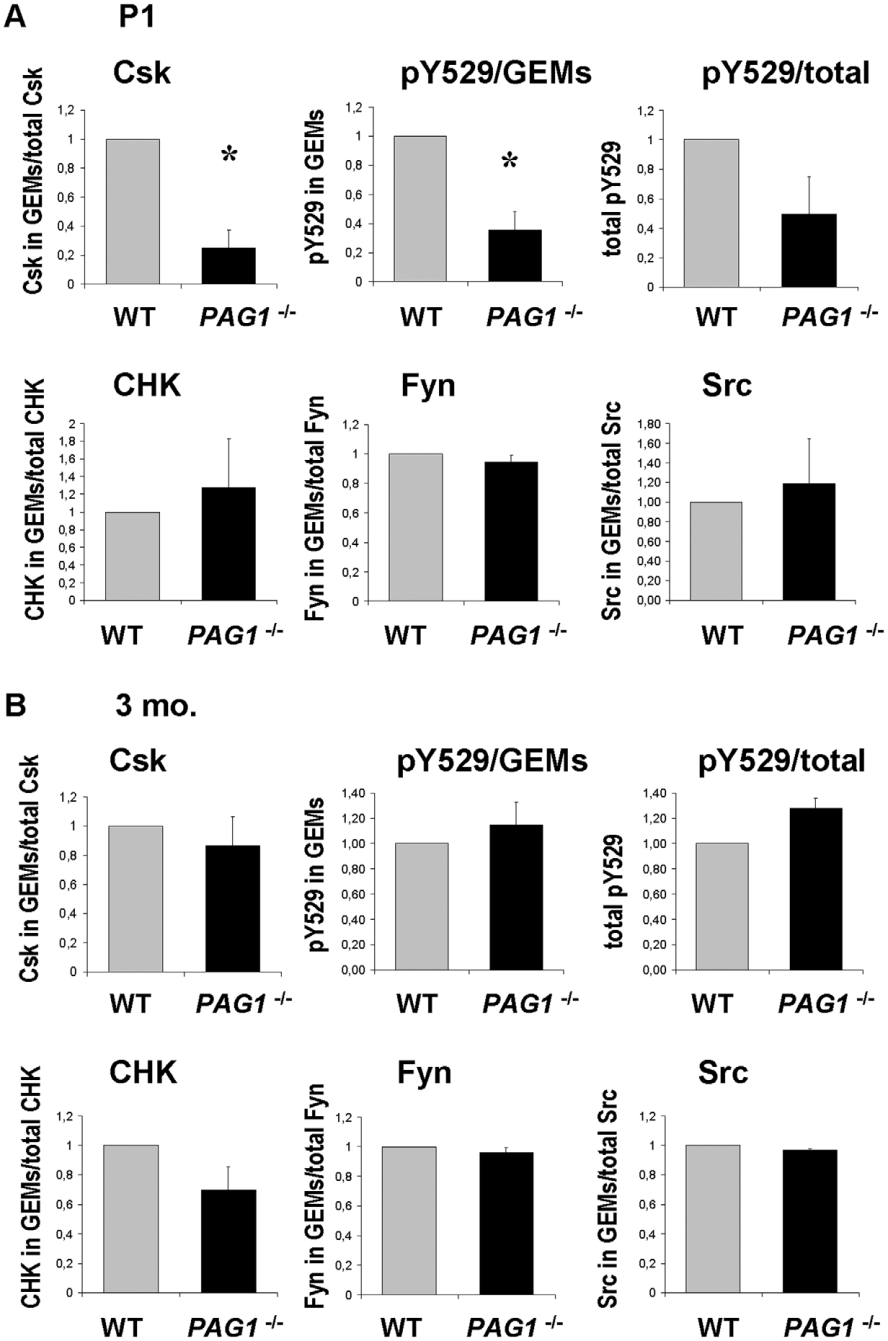
Quantification of kinases in GEM gradients. The kinase bands in each fraction represented in Fig. 5 were quantified from three independent experiments for A) the P1 and B) the 3 month time point. The summative signal from GEM fractions in relation to the total signal is presented as ratio to wildtype for each kinase. GEM containing fractions were determined by choleratoxin staining. For phosphotyrosine 529 the total signal in GEMs and the total signal on the blot are given in separate graphs, as most of the signal localizes to GEM fractions and the total amount of pY529 is reduced in *PAG1* ^-/-^ mice (see also Fig. 6). Given are the means +/− SEM. •p<0.05, Student's t-test, all other pairs did not show significant differences.

To analyze PAG function, we generated *Pag1*-knockout mice by homologous recombination as described in the Materials and Methods (Fig. S1). *Pag1*-knockout mice are negative for the PAG protein (Fig. 5 and Fig. S3). Note that additional bands appear in Western blots from 3 month old wild type and *Pag1* ^-/-^ brains that are recognized by our antiserum (Ig452), however these bands are peptide-unspecific (Fig. 6B) and thus do not represent PAG. Using an antiserum against the N-terminal peptide (P18, 1–18) and a different antiserum against the cytoplasmic domain (Rabbit anti-PAG, 97-432) we could also not detect a truncated protein (Fig. S3). *Pag1* ^-/-^ mice had no alteration in birth weight, showed no obvious neurological phenotype, and appear to live to normal age with the oldest animal in our colony now reaching 2.5 years.

PAG predominantly localizes to lipid rafts in lymphocytes and postnatal mouse brain [14], [20]. We confirm that the majority of PAG is found in the lipid raft fractions in postnatal brains (Fig. 5A). In adult brains, lipid rafts are consistently found one fraction lower than at P1, probably due to the high lipid content of myelinated brain. However, rafts can clearly be isolated, demonstrated by the binding of cholera toxin to GM1-lipids. Similar to P1, PAG strongly associates with the lipid raft fractions (Fig. 5B).

The Src family kinases Fyn and Src are found both inside and outside of lipid rafts, as previously described [20]. Interestingly, while the majority of Fyn is localized to the lipid rafts in both newborn and adult brain, only a minor proportion of Src is raft-associated in newborn murine brain. In adult brain the distribution of Fyn and Src is similar. Notably, given the association of PAG with Fyn or Src in wild type-animals (Fig. 3), neither the distribution of Fyn nor Src is altered in the brains of *Pag1*-deficient mice (Fig. 5A and B, 6A). We next investigated whether the localization of Csk is altered in the brains of *Pag1*-knockout mice. We found that the amount of Csk within GEMs is reduced to 25 % of wildtype in P1 *Pag1* ^-/-^ brains. (Fig. 5A, 6A, p<0,05). In agreement with this finding, the phosphorylation of the inhibitory tyrosine Y529 in Src/Fyn within GEMs, which is dependent upon Csk or CHK, is reduced to 35% in postnatal (P1) brains of *Pag1*-deficient mice (Fig. 5A, 6A, p<0.05). Note that the majority of the pY529 signal is found within the lipid rafts in both postnatal and adult animals. However, the amount of Csk that can be detected in lipid rafts of adult wild type mice is minute. Interestingly, the amount of lipid raft-associated Csk does not appear to differ in adult *Pag1*-knockouts (Fig. 5B and 6B). Phosphorylation of Y529, which is generally much lower than in newborn mice, is also unchanged in lipid rafts of adult knockout animals (Fig. 5B and 6B). Despite its association to PAG in wild type mice, the distribution of CHK is not altered in either newborn or adult *Pag1* ^-/-^ mice (Fig. 5A, B, and 6A, B).

**Figure 7:**
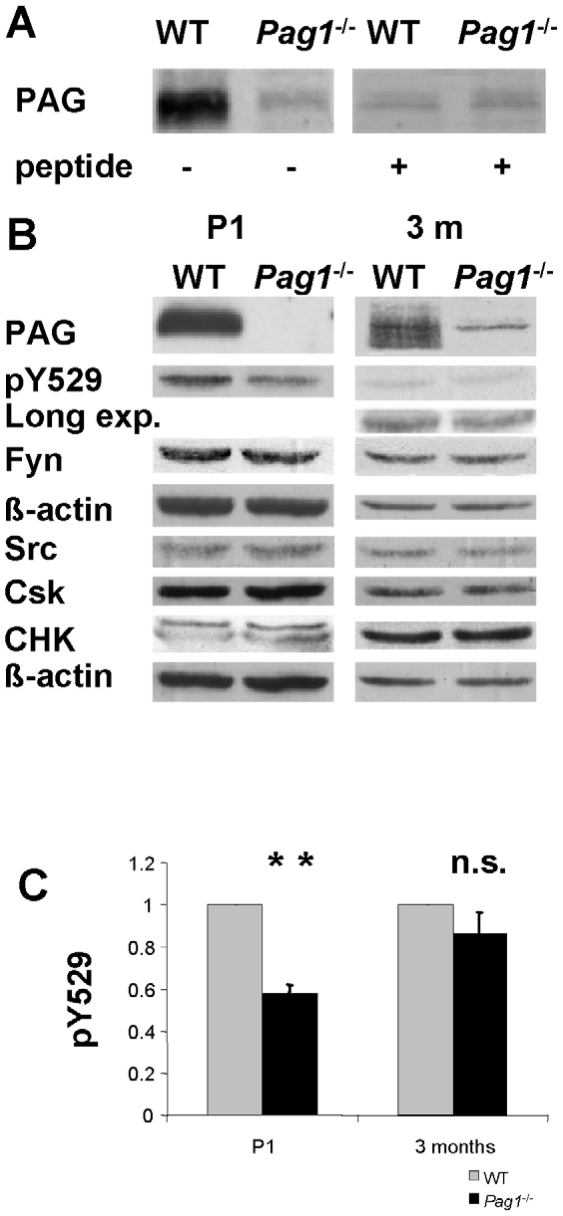
Decreased phosphorylation of Src-kinase negative-regulatory tyrosine 529 in P1- *Pag1* ^-/-^ mice. For P1-material, protein extracts were prepared from separate newborn litters of time-mated *Pag1* ^-/-^ mice and wild type C57Bl6 mice. For 3 months-material, protein extracts were prepared from adult *Pag1* ^-/-^ and wild type littermates of *Pag1* ^+/-^ crossings. **A**) Incubation with anti-PAG-antiserum Ig452 detects a band at 75-80 kDa in brain extracts of 3 month old *Pag1* ^-/-^ mice. Co-incubation with the antigenic peptide abolished the specific PAG-band in WT mice, but left the additional band unchanged in both WT and *Pag1* ^-/-^ extracts, indicating that this band is not peptide-specific. **B**) Equal concentrations of protein from each sample were analyzed by immunoblotting for PAG (Ig452), Csk, CHK, pY529, Fyn, and Src. Actin staining served as loading control. **C**) Phosphorylation of the negative regulatory tyrosine (pY529) was quantified in different experiments and is presented as the ratio to wild type. Mean and SEM are given for n = 4 (P1) and n = 6 (3 months) independent experiments. ** p<0.01, n.s. not significant, Student's t-test.

To compare total expression levels and tyrosine phosphorylation, we investigated whole brain lysate of wild type and *Pag1* ^-/-^ mice (Fig. 7). *Pag1*-deficiency (Fig. 7A) did not lead to a change in the expression of Csk and CHK or the Src-kinases Fyn and Src in either postnatal or adult brain (Fig. 7B). In postnatal brain however, phosphorylation of the inhibitory tyrosine in SFKs was reduced by 42% in knockout mice (analyzed over 4 independent experiments), paralleling the reduction of Csk in the lipid raft fractions (Fig. 7B and C). In brain lysate of adult mice, a trend to a reduction of around 14% in the pY529 signal is observed, which is not significant on statistical testing (p = 0.18), indicating at least a partial compensation for the lack of PAG during brain maturation (6 independent experiments; Fig. 7C).

### PAG differentially modulates Src kinase activity in postnatal and adult brain

**Figure 8:**
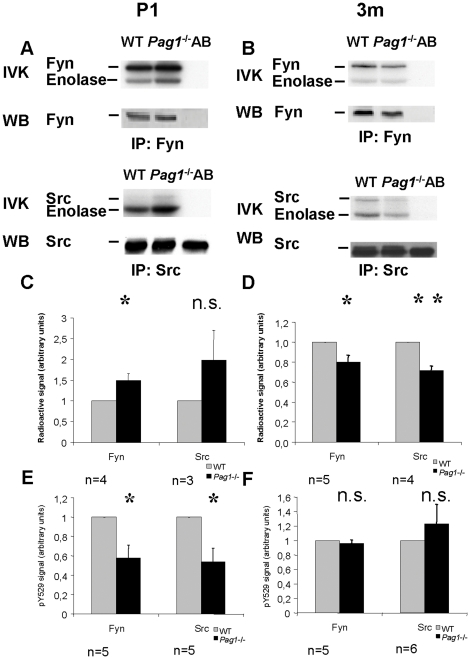
Src kinase activity is altered in both postnatal and adult *Pag1* ^-/-^ brain. GEM-containing fractions (identified via cholera toxin subunit B-binding) of **A**) postnatal or **B**) adult wild type and *Pag1* ^-/-^ brains were pooled, 10% was retained for a Western blot control, the rest was split into two samples, from which Fyn and Src, respectively, were precipitated. Precipitates were again split in halves and subjected to either *in vitro* kinase assay (upper panels) or Western blot (lower panels) which controlled for equal amounts of kinase precipitated. **C**) and **D**) Kinase activities are presented as ratio to wild type. **E**) and **F**) Phosphorylated Y529 was quantified in the Western blot controls of Fyn and Src immunoprecipitates and normalized to the amount of precipitated SFK. Given are the means +/- SEM, n =  number of independent experiments. p<0.05, ** p<0.01, n.s. not significant, Student's t-test.

**Figure 9:**
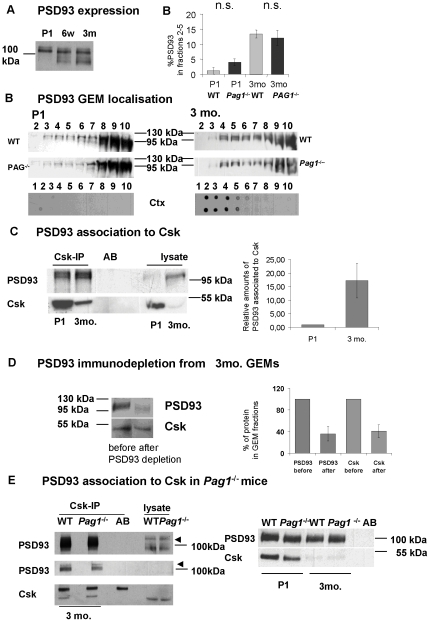
Developmental expression and Csk-binding of PSD93. **A**) Whole brain lysates from WT mice of the indicated ages were Western blotted and stained for PSD93. **B**) Brains of P1 wild type and *Pag1* ^-/-^ mice and 3 month old WT/ *Pag1* ^-/-^ littermate pairs were lysed at concentrations of 3 ml/g and 4.5 ml/g, respectively, in GEM-lysis buffer and subjected to sucrose-density centrifugation. Fractions were analyzed by Western blotting and staining for PSD93. Lipid raft fractions were identified by binding of cholera toxin subunit B (Ctx) binding to GM1-lipids in a dot blot from the fractions. **C**) Densitometric quantification of the PSD93 signal in GEM-fractions as percentage of the total PSD93-signal. For both time points, the PSD93 signal in fractions 2-5 was expressed as a ratio to control. This most likely overestimates the lipid raft localized material in P1, as only fraction 2 and 3 contain rafts (n = 3 independent experiments for each time point) **D**) Csk-immunoprecipitates from whole brain lysates of WT mice (ages as indicated), stained for PSD93 and Csk. Densitometric quantification of the relative amount of PSD93 bound to Csk in brains of P1 and 3 month old mice (n = 3 independent experiments) **E**) Csk-immunoprecipitates from whole brain lysates of 3 month old WT and *Pag1* ^-/-^ mice stained for PSD93 and Csk. Two exposures are given for the PSD93-signal in the left panel. Comparison of whole brain lysates of 3 month old WT and *Pag1* ^-/-^ to P1. Given are the means +/- SEM, n.s. not significant, Student's t-test.

To test whether the decreased phosphorylation of the inhibitory tyrosine is associated with increased kinase activity in postnatal *Pag1* ^-/-^ mice, we next performed *in vitro* kinase assays for Fyn and Src from whole brain lysates. No difference was observed (data not shown). However, as PAG is predominantly found within the rafts, we hypothesized that it might exert its regulatory function only on a subset of SFKs. Thus, when the Src kinases were precipitated from the lipid rafts, *Pag1* ^-/-^ mice showed an increase in both auto- and substrate phosphorylation. The basal activity of Fyn was increased by 50% (p<0,05), Src activity showed a similar trend, however the result was not significant (p = 0.31) (Fig. 8A, C). This corresponded well to a 40% decrease of Y529 phosphorylation, which was significant in both Fyn- and Src-immunoprecipitates (Fig. 8E). To our surprise, adult *Pag1* ^-/-^ brains showed the reverse phenotype with a decrease in basal Fyn and Src kinase activity of −20% and −28%, respectively (p<0.05 and p<0.01, resp., Fig. 8B, D), despite normal Csk-distribution and nearly normal Y529 phosphorylation (Fig. 8F), indicating a net change of 70–80% in kinase activity within three months. The phosphorylation of other regulatory tyrosines (Y418, Fyn Y215) was not altered in adult *Pag1* ^-/-^ brains (data not shown). As a possible substitute for PAG in brain we explored LIME, another lipid-raft-associated Csk-binding phosphoprotein with adaptor function [28], [29]. Although in situ-hybridisation data suggest that LIME is indeed expressed within this organ (http://www.brain-map.org), we were unable to confirm protein expression in mouse brain due to strong cross-reactivity of our antibodies/-sera. However, LIME is unlikely to function as the main compensator for the lack of PAG as *Lime1* ^-/-^/ *Pag1* ^-/-^ double knockout mice are viable and fertile (J. Lindquist, unpublished observation). Another lipid-raft –bound Fyn-substrate, which phospho-dependently binds Csk, is PSD93, an important component of neuronal postsynaptic densities [30]. Belonging to the family of membrane-associated guanylate kinases, PSD93 also functions as an adaptor. We showed increasing expression in the first postnatal weeks (Fig. 9A). Furthermore, in adult brain a greater proportion of the cellular PSD93 is localized in lipid rafts of WT and *Pag1* ^-/-^ mice (Fig. 9B). At 3 months a much stronger association to Csk compared to P1 could be found (Fig. 9C). After repeated immunoprecipitation of PSD93 from GEMs, Csk was also reduced (Fig. 9D), indicating that PSD93 binds Csk within the GEMs and can recruit Csk to the membrane possibly substituting for PAG in the brains of older mice. Note, PSD93 can be co-precipitated with Csk in abundance, while the Csk-PAG-association can in our hands only be shown via immunoprecipitating PAG, not vice versa. Therefore, one roles of PSD93 in adult WT mice might be the recruitment of Csk, and the amount of PSD93 that could be additionally bound to Csk to substitute for PAG in adult *Pag1* ^-/-^ mice may not be detectable (Fig. 9E).

### SH2-dependent binding of Fyn to PAG may lead to an active conformation

The surprising finding that SFK activity is decreased in the absence of PAG lead us to hypothesize that one function of PAG may be to keep Src kinases in an open conformation. Thus we investigated the Fyn binding site in PAG. We constructed several N-terminally tagged STOP-mutants of PAG (Fig. S4) and expressed them in HEK 293T cells (Fig. S3). Membrane targeting of the STOP mutants is shown in Fig. S5. In vitro kinase assays demonstrated that Fyn kinase activity was associated with WT PAG as well as with the STOP3 and STOP4 constructs. However, kinase activity was not seen with either STOP1 or STOP2, which lack the N-terminal proline rich region (Fig. S4B). Previously, the PAG-Fyn-association was proposed to be constitutive, mediated by the Fyn-SH3 domain [14], however, a contribution of the SH2-domain was suggested [14], [31]. To confirm this result, Fyn immunoprecipitates were performed from transfected cells and probed with anti-Flag (Fig. S4C). Surprisingly, Fyn binding is observed with all mutants, except STOP 1, indicating that Fyn can also bind to Y105. Together these results identify the membrane proximal proline-rich region and Y105 as potential binding sites for the SH3 and SH2 domains of Fyn, respectively. Binding of the Fyn SH2-domain to PAG may prevent the intramolecular binding of the C-terminus, when phosphorylated at the inhibitory tyrosine. By this mechanism, PAG may prevent SFKs from adapting the closed inactive conformation.

## Discussion

Src family kinases regulate multiple processes in brain development and function, such as neuronal survival, differentiation, migration, and axonal path finding, as well as synaptic plasticity. Here we describe the transmembrane adaptor PAG as a novel component in the regulatory circuits of Fyn and Src activity in the brain. We identify lipid rafts as the platform where negative regulatory tyrosine phosphorylation of SFK takes place and show that the correct localization of regulatory components to lipid rafts is a prerequisite for proper SFK-signaling in the brain. We demonstrate differential PAG expression patterns in postnatal and adult brain and show that mechanisms of SFK regulation differ dependent on maturation.

Within the immune system, PAG has been established as an important negative regulator of Src kinase activity [14], [20], [32]. Therefore, *Pag1*-knockout mice were predicted to have a severe phenotype, comparable to *Csk*-deficient mice [33]. Surprisingly, they turned out to be viable and healthy [34], [35]. Having found no difference in lymphocyte development and function, both groups concluded that in their system PAG was dispensable as a regulator of SFKs. However, our recent study employing RNA interference to down-regulate PAG expression clearly demonstrates that in the absence of PAG, Src-kinases are hyperactive, thus suggesting that the *Pag1*-knockout mice had developed a compensatory mechanism [23]. Here we analyze a third *Pag1*-knockout strain, focusing on a different organ with abundant PAG-expression, the brain. Brain provides an advantageous experimental system as firstly, Csk is absolutely required for brain development. Mice deficient for Csk show a dramatic increase in SFK activity and are embryonic lethal due to neural tube defects and massive neuronal apoptosis [12], [13]. Secondly, postnatally Csk-expression declines sharply, suggesting that other mechanisms of SFK regulation gain importance. In postnatal brain we can demonstrate the expected phenotype for *Pag1*-deficiency, i.e. reduced phosphorylation of the inhibitory tyrosine (Y529) in Fyn and Src correlating with a marked reduction of Csk-recruitment into lipid rafts, which is most likely enhanced by the lack of stimulation in Csk activity that normally results from PAG-binding [36]. In agreement with our study, Xu et al. showed reduced Csk-recruitment to lipid rafts, but did not explore the effects on regulatory tyrosine phosphorylation [35]. Dobenecker et al. found no change in Csk distribution [34]. Although both studies analyzed similar cells, methodological differences, e.g. in lipid raft preparation, may account for the dissimilar results. We demonstrate that SFKs phosphorylated at Y529 reside predominantly within the lipid rafts, indicating that the enzyme responsible for phosphorylating this tyrosine, Csk or CHK, needs to be recruited to this membrane compartment. The functional importance of the lipid raft localization of Csk is illustrated by our recent study of a mutant PAG molecule lacking the palmitoylation motif, which localizes to the membrane, but not to lipid rafts. Here, Csk is accumulated outside of rafts resulting in a prolonged Src kinase activation upon stimulation and an enhanced migration of these cells in response to chemokine [37]. Now we show, in postnatal brain, that the decreased recruitment of Csk into lipid rafts in *Pag1* ^-/-^ mice results in a 50% increase in SFK activity within lipid rafts, which correlates well with our observation of a 40% decrease in the phosphorylation of the inhibitory tyrosine. The demonstrated increase in kinase activity may appear rather small compared to the *Csk*-knockouts where 4-14 fold increases in specific activity were found. However, these values were counter-balanced by a dramatic downregulation of the expression levels of Fyn, Src, and Lyn. The resulting net increase in active kinase nevertheless proved deleterious [12], [13], emphasizing the importance of tightly regulating Src-kinase activity. In our *Pag1* ^-/-^ mice neither the expression levels of Fyn and Src nor their lipid raft association were altered, thus exposing the cells to a 50% net increase in SFK activity. Interestingly, mice deficient in protein tyrosine phosphatase α (PTPα), which counter-regulates Csk activity, show a 50% increase in the phosphorylation of Y529 and a 50–60% decrease in Src and Fyn activity [38]. In the absence of gross morphological alterations in the brain, these mice still present with a subtle neurological phenotype including reduced spatial learning, locomotor activity, and anxiety [39]. Considering that Csk inhibits all Src kinases, a 50% increase of basal SFK-activity particularly in GEMs may be of similarly subtle phenotypic consequence.

In P1 brains, the function of PAG is closely connected to Csk recruitment. We show that in adult brain although PAG is abundant, Csk concentrations are minimal and very little Csk is bound to PAG. We demonstrate the binding of the second negative-regulatory tyrosine kinase, CHK, which is more strongly expressed postnatally, to PAG, pointing to a broader role for PAG in the regulation of SFK in the central nervous system. The observation of a phospho-dependent interaction of CHK with PAG [40] supports our results. However, a CHK association to PAG in brain may also be indirect, e.g. via the association of CHK to SFK or transmembrane receptors [41]. The finding of unchanged expression levels of CHK and unaltered distribution within lipid rafts in our *Pag1*-deficient mice demonstrates firstly, that CHK does not compensate for the reduced recruitment of Csk and secondly, that PAG is dispensable for CHK localization to this membrane compartment in brain.

In adult brain, PAG expression overlaps with the localization reported for both Fyn and Src, specifically the brain-specific isoform pp60 c-src(+), in the hippocampus and the dentate gyrus, as well as in the olfactory bulb and the cerebellum. Interestingly, the specific concentration of PAG in the septal nuclei, habenula, and pineal gland is also shared with pp60 c-src(+) [42]. Furthermore, we could show for the first time the binding of Fyn and Src to PAG in primary tissue. The shared expression pattern and close association of PAG and SFKs in a situation where little Csk is expressed lead us to explore whether PAG has a role for the regulation of SFKs beyond Csk-recruitment.

In contrast to the situation in P1 postnatal brains, no alteration in the subcellular distribution of Csk was detectable within the brains of adult *Pag1* ^-/-^ mice. Consequently, there was also no significant decrease in Y529 phosphorylation of Fyn or Src. There was no counter-regulation in the level of Csk or CHK expression. The surprising finding of a 20–30% decrease of Fyn and Src activity in brains of *Pag1*-deficient mice indicates that compensatory mechanisms are strengthened during brain maturation. The lipid raft-resident adaptor PSD93 may in part substitute for PAG in adult brain. We show a strong increase in PSD93 expression, lipid raft association, and binding to Csk postnatally. We demonstrate that PSD93 binds Csk within lipid rafts and that by immunodepleting PSD93, Csk can also be markedly reduced, indicating that PDS93 can recruit Csk to lipid rafts. Other candidates for Csk recruitment to lipid rafts in adult brain are paxillin [43], [44], caveolin-1 [45], and ZO-1/2 [46].

Restored Csk-recruitment however, does not explain the decrease in Src and Fyn activity observed in the brains of adult *Pag1* ^-/-^ mice. We do not observe increased Y529 phosphorylation, therefore an upregulation of Csk activity, e.g. by cAMP dependent protein kinase (PKA)-induced phosphorylation of Csk [47] or any other PAG-independent role of Csk, is unlikely to contribute to the compensatory mechanism. Unaltered Y529 phosphorylation in adult knockout mice also excludes compensation through PTPα. Counter-regulation affecting Y418 within the activation loop may be postulated. However, in accordance with the previous analysis of *Pag1* ^-/-^ mice [34], we did not find any alteration in the phosphorylation levels of this tyrosine residue. Greatly enhanced kinase activity can also follow phosphorylation of Src Y215, particularly in the presence of inhibitory tyrosine phosphorylation [10]. We have shown recently that not only increased Src, but also increased Fyn kinase activity is associated with Y215 phosphorylation [23]. Again, we did not find any difference in the levels of pY215 between the brains of wild type and *Pag1* ^-/-^ mice. Finally, SFK activity can also be enhanced by stabilizing an open/active conformation of the Src kinases [48], [49]. We show here that the binding of Fyn to PAG involves the first proline-rich region as well as Y105 in PAG, thus confirming the findings of Solheim et al. [50]. This group recently demonstrated that PAG-binding enhances Fyn kinase activity *in vitro* and suggested that the binding of the Fyn SH2 domain to PAG prevents the intramolecular binding with its phosphorylated C-terminus, thereby maintaining an open/active conformation. Additionally, the ‘free’ phosphorylated C-terminus may then function as a binding site for other SH2-domain-containing proteins, such as STAT3, as we have recently shown [51]. Support for a positive regulatory function of PAG is also provided by the study of Davidson et al. [32]. In transgenic animals overexpressing a mutant form of PAG lacking the Csk binding site, SFK activity following TCR stimulation is enhanced. The phenotype of our adult *Pag1* ^-/-^ mice and the small regional overlap in expression of PAG and Csk supports such a Csk-independent positive regulatory role for PAG in adult brain. PAG has been shown to influence SFK signaling independent of Csk by sequestering SFKs into lipid rafts [52] and by altering the levels of GM1 in rafts [53]. In our *Pag1* ^-/-^ mice the lipid raft localization of Fyn or Src was not altered, however, the recruitment of other signaling components into the lipid rafts should be investigated. Apart from forming a binding site for Csk, PAG possesses nine other tyrosine residues that are potential binding sites for adaptors such as Shc and Grb2, as well as enzymes like Lck, Fyn, Lyn, ZAP-70, Shp-1, Shp-2, PI3K, RasGAP, and Vav [14]. Furthermore, we have recently identified a multiprotein complex containing RasGAP and Sam68, which links PAG to both the regulation of Ras [23] and possibly to nuclear-dendritic feedback [54].

While our biochemical analysis focused on quantitative changes in whole brain extracts, further studies need to delineate cell-type-specific effects of PAG deficiency and the consequences for brain function, e.g. for the processing of strong emotions, learning, and memory, for which those structures with high expression levels of PAG, septum, habenula, and hippocampus, are central.

## Supporting Information

Figure S1 **Antibodies against PAG, Fyn and Src work phospho-independently.** Jurkat T cells were either left unstimulated, incubated with 10 µM PP2, a Src kinase inhibitor, for 30 min to reduce phosphorylation, or treated with pervanadate to maximize phosphorylation. Equal cells numbers were lysed and then protein concentrations determined to ensure equal loading. The cell lysates were the separated by SDS-PAGE and transferred onto nitrocellulose membranes. Western blots were performed with the indicated antibodies (PAG[Ig452], Rabbit anti-PAG, Fyn-02, and Src [clone GD11]). Additionally, pan-phosphotyrosine [4G10] staining was performed to verify successful treatment of the cells, Grb2 was included as the loading control.
(TIF)

Figure S2 **Generation of** ***Pag1*** **-knockout mice.** **A**) Structures of the wild-type gene and mutant gene. **B**) PCR results using primers indicated in *A* show correct integration at the 5′ and the 3′ sites. **C**) Restriction digestion of DNAs from wild type (WT) and mutant BACs. **D**) Fluorescence *in situ* hybridization analysis of cell lines confirms successful targeting in clones 7 and 35.
(TIF)

Figure S3 **Western blots with Rabbit anti-PAG and anti-PAG [P18] confirm that no PAG protein is expressed in** ***Pag1*** **-knockout mice.** Whole brain lysates from P1 wild type and *Pag1* ^-/-^ mice were subjected to sucrose-density centrifugation. Fractions were analyzed by Western blotting. PAG was detected using the Rabbit anti-PAG (97-432) antiserum or the anti-PAG [P18] serum recognizing the N-terminal part of the protein. No full length or truncated PAG protein could be detected. The asterisk indicates the gel front, the arrowheads point to unspecific bands in WT and KO, which run at a different MW and in the heavy fractions, contrary to PAG.
(TIF)

Figure S4 **Fyn binding site in PAG.** **A)** Truncation mutants generated from N-terminally Flag-tagged PAG. The black triangle represents the epitope tag, the transmembrane region (TM) is indicated with an unfilled box, the palmitoylation motif is indicated with asterisks, and filled boxes indicate the proline rich regions (PRO). The numbers represent the location of tyrosine residues with PAG. **B)** Autoradiograph from *in vitro* kinase assays performed on Flag-immunoprecipitates from transfected HEK 293T cells. The Fyn immunoprecipitate is included as a control. **C)** Fyn immunoprecipitates from HEK 293T cells transfected with the indicated constructs. Western blots were stained with rabbit-anti-Flag. The bands at 55 and 25 kDa are the heavy and light chains of the immunoprecipitating antibody. **D)** Expression of the PAG constructs. Rabbit-anti-Flag staining of post-nuclear cell lysates from HEK 293T cells transfected with the indicated constructs. Apparent molecular weight (kDa) is indicated to the right.
(TIF)

Figure S5 **Membrane localisation of the PAG constructs.** Jurkat T- cells transfected with the indicated constructs were stained with rabbit-anti-Flag/donkey-anti-rabbit-FITC and mouse-anti-CD29/donkey-anti-mouse-Cy5 antibodies.
(TIF)
