# Supplementary figures and images for: Phosphoprotein Associated with Glycosphingolipid-Enriched Microdomains Differentially Modulates Src Kinase Activity in Brain Maturation
Source: PLoS One. 2011 Sep 6;6(9):e23978. doi: 10.1371/journal.pone.0023978 (PMC3167820; doi:10.1371/journal.pone.0023978)

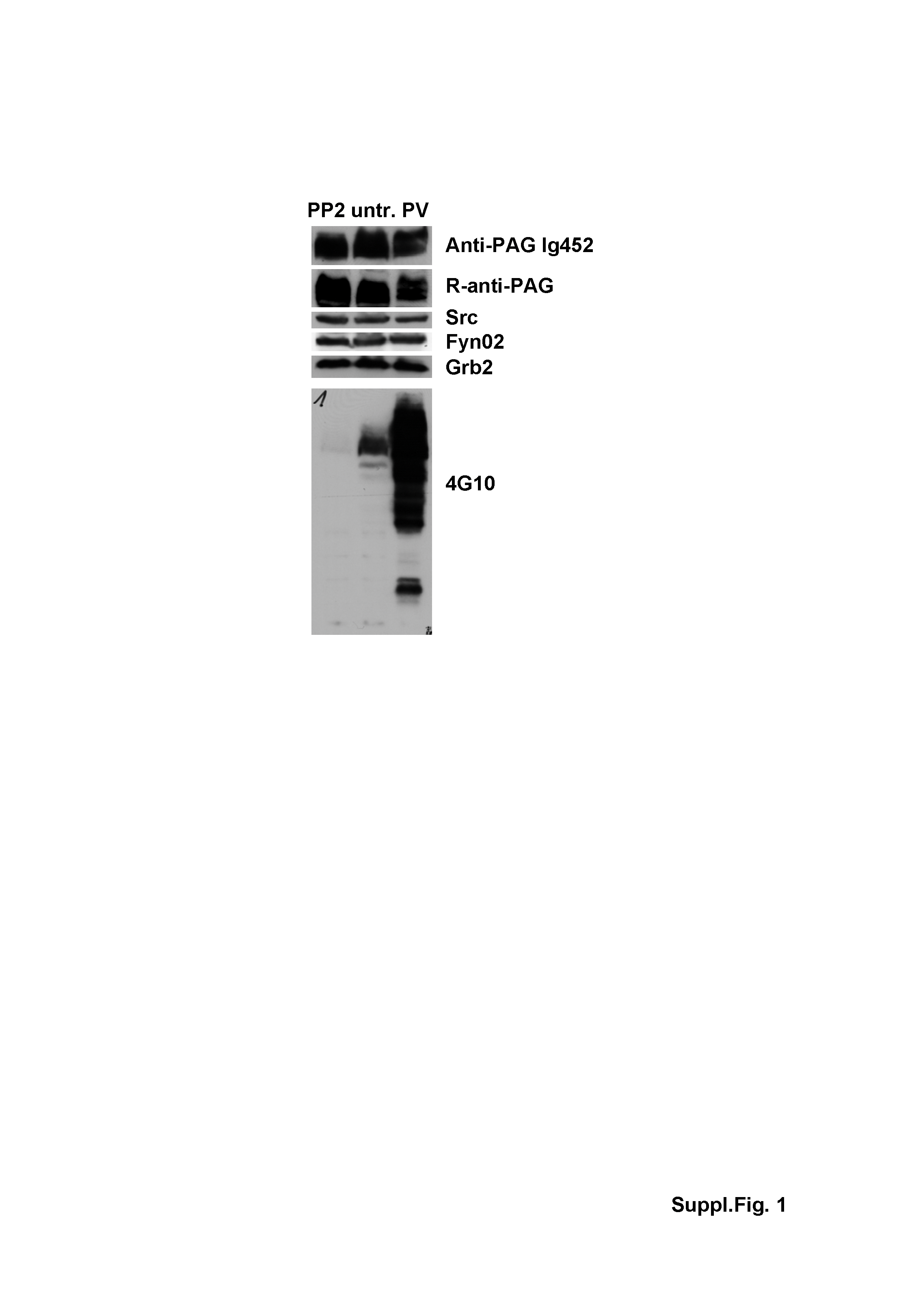

Supplement: Figure S1 — Antibodies against PAG, Fyn and Src work phospho-independently. Jurkat T cells were either left unstimulated, incubated with 10 µM PP2, a Src kinase inhibitor, for 30 min to reduce phosphorylation, or treated with pervanadate to maximize phosphorylation. Equal cells numbers were lysed and then protein concentrations determined to ensure equal loading. The cell lysates were the separated by SDS-PAGE and transferred onto nitrocellulose membranes. Western blots were performed with the indicated antibodies (PAG[Ig452], Rabbit anti-PAG, Fyn-02, and Src [clone GD11]). Additionally, pan-phosphotyrosine [4G10] staining was performed to verify successful treatment of the cells, Grb2 was included as the loading control. (TIF) [file pone.0023978.s001.tif]

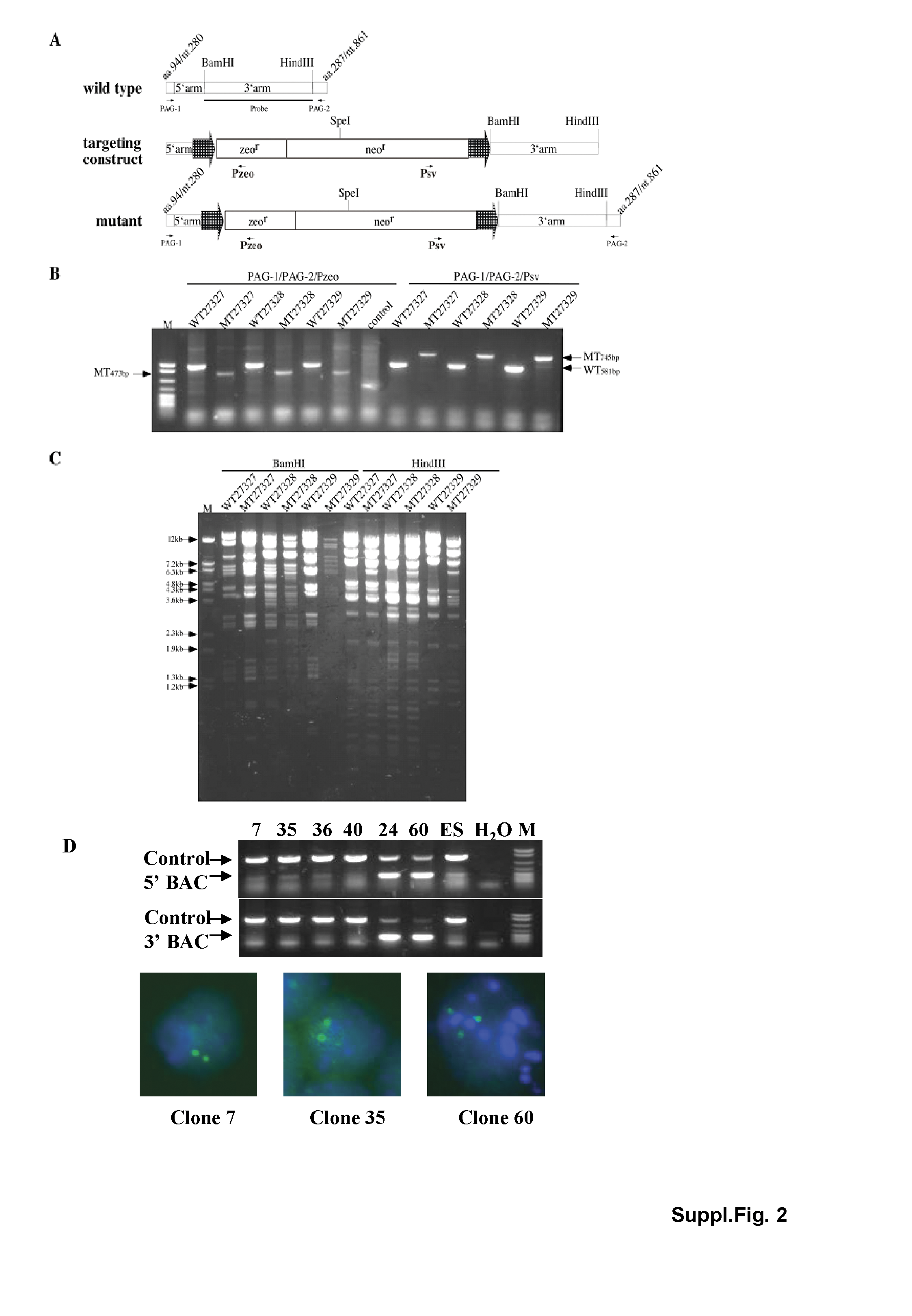

Supplement: Figure S2 — Generation of Pag1 -knockout mice. A) Structures of the wild-type gene and mutant gene. B) PCR results using primers indicated in A show correct integration at the 5′ and the 3′ sites. C) Restriction digestion of DNAs from wild type (WT) and mutant BACs. D) Fluorescence in situ hybridization analysis of cell lines confirms successful targeting in clones 7 and 35. (TIF) [file pone.0023978.s002.tif]

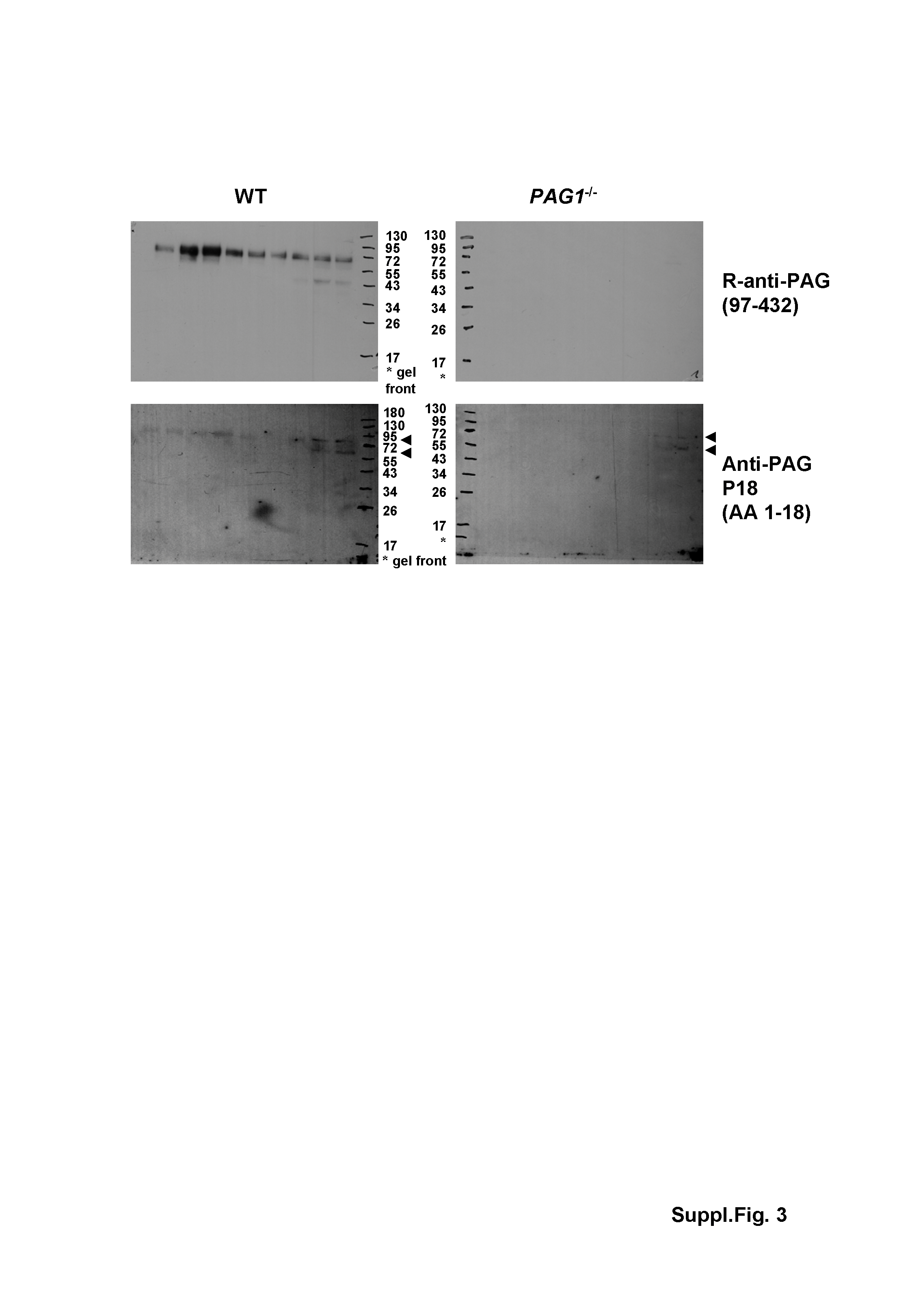

Supplement: Figure S3 — Western blots with Rabbit anti-PAG and anti-PAG [P18] confirm that no PAG protein is expressed in Pag1 -knockout mice. Whole brain lysates from P1 wild type and Pag1 -/- mice were subjected to sucrose-density centrifugation. Fractions were analyzed by Western blotting. PAG was detected using the Rabbit anti-PAG (97-432) antiserum or the anti-PAG [P18] serum recognizing the N-terminal part of the protein. No full length or truncated PAG protein could be detected. The asterisk indicates the gel front, the arrowheads point to unspecific bands in WT and KO, which run at a different MW and in the heavy fractions, contrary to PAG. (TIF) [file pone.0023978.s003.tif]

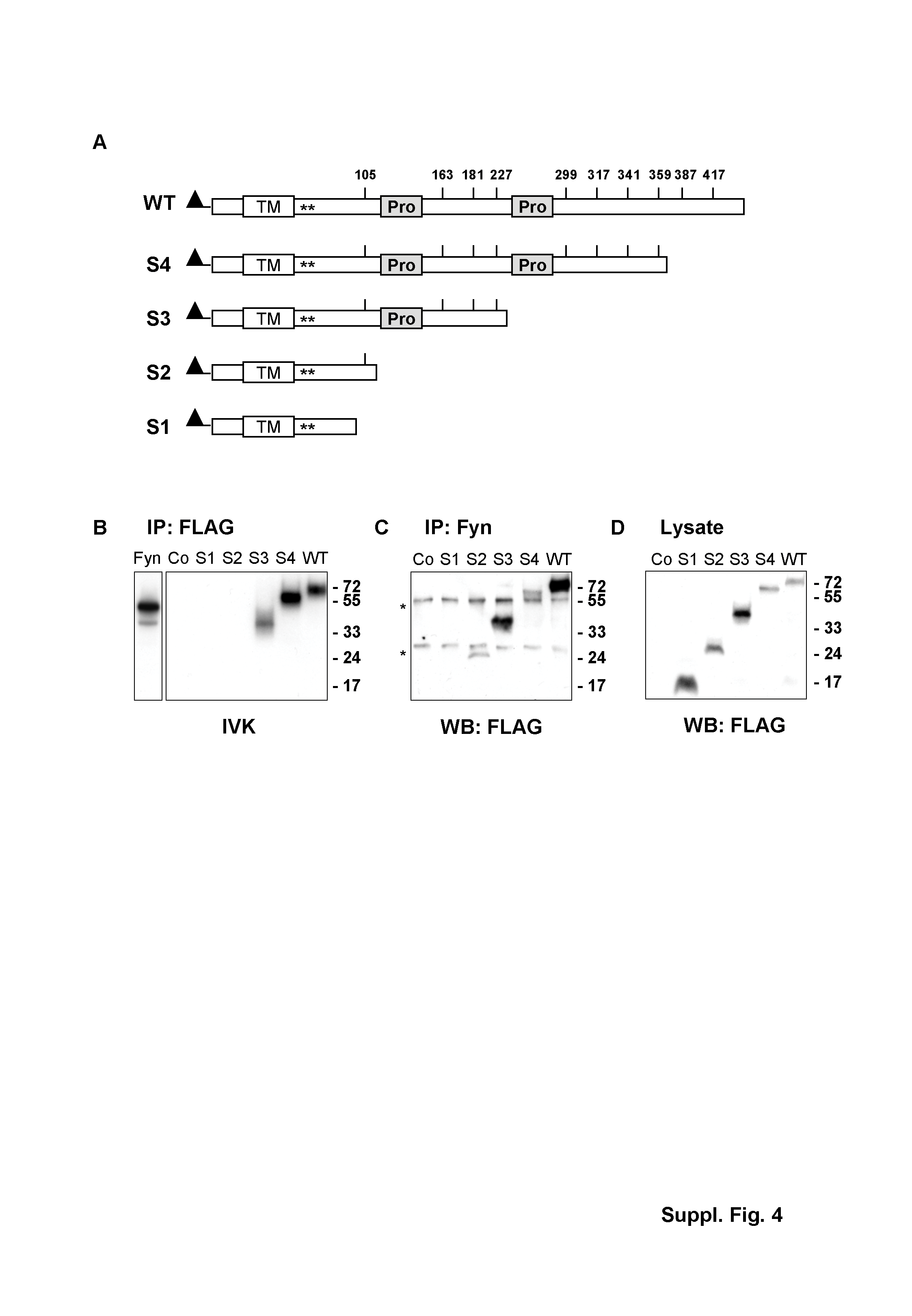

Supplement: Figure S4 — Fyn binding site in PAG. A) Truncation mutants generated from N-terminally Flag-tagged PAG. The black triangle represents the epitope tag, the transmembrane region (TM) is indicated with an unfilled box, the palmitoylation motif is indicated with asterisks, and filled boxes indicate the proline rich regions (PRO). The numbers represent the location of tyrosine residues with PAG. B) Autoradiograph from in vitro kinase assays performed on Flag-immunoprecipitates from transfected HEK 293T cells. The Fyn immunoprecipitate is included as a control. C) Fyn immunoprecipitates from HEK 293T cells transfected with the indicated constructs. Western blots were stained with rabbit-anti-Flag. The bands at 55 and 25 kDa are the heavy and light chains of the immunoprecipitating antibody. D) Expression of the PAG constructs. Rabbit-anti-Flag staining of post-nuclear cell lysates from HEK 293T cells transfected with the indicated constructs. Apparent molecular weight (kDa) is indicated to the right. (TIF) [file pone.0023978.s004.tif]

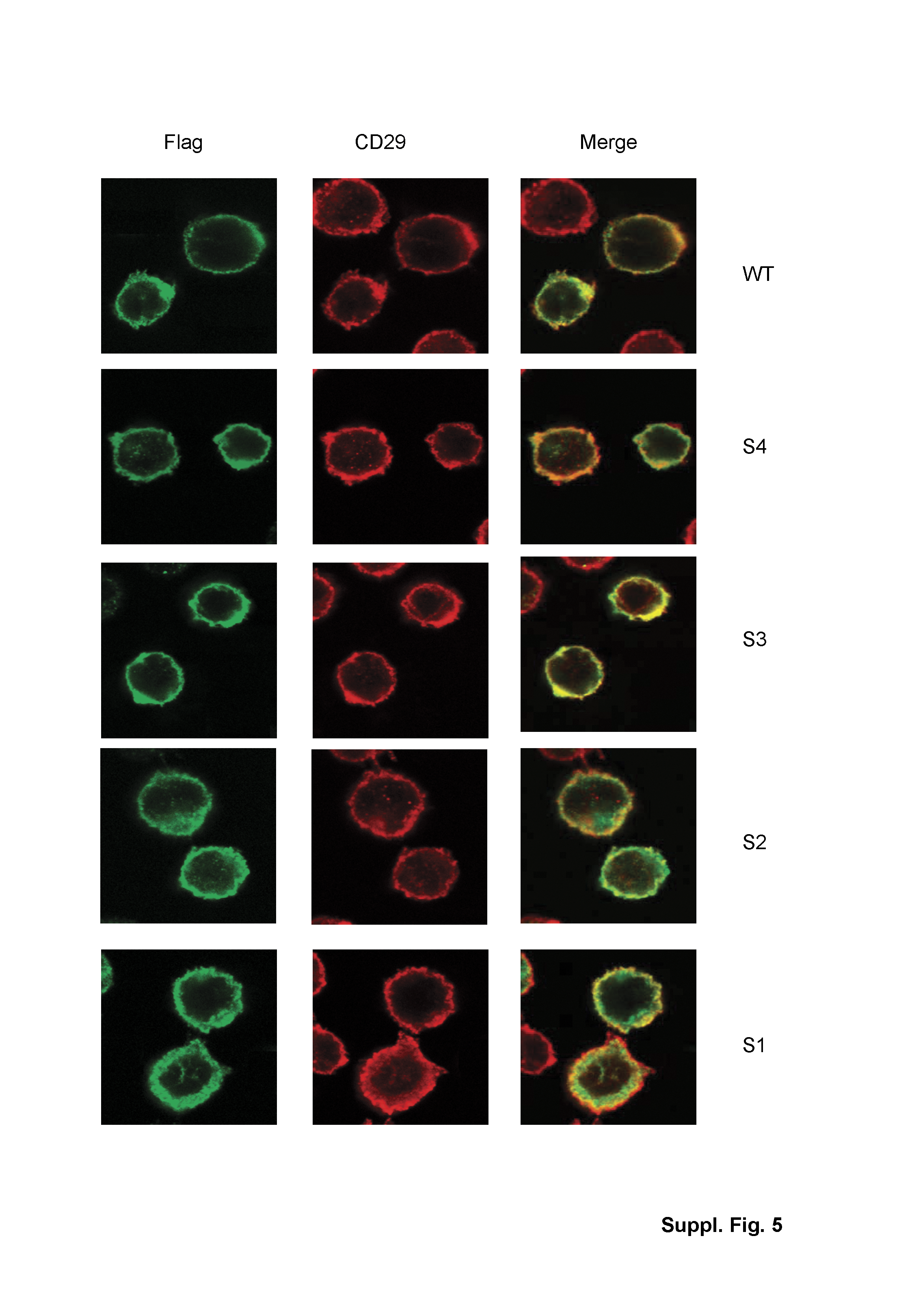

Supplement: Figure S5 — Membrane localisation of the PAG constructs. Jurkat T- cells transfected with the indicated constructs were stained with rabbit-anti-Flag/donkey-anti-rabbit-FITC and mouse-anti-CD29/donkey-anti-mouse-Cy5 antibodies. (TIF) [file pone.0023978.s005.tif]
